# PAS-cal: a Generic Recombinant Peptide Calibration Standard for Mass Spectrometry

**DOI:** 10.1007/s13361-014-0902-3

**Published:** 2014-05-28

**Authors:** Joscha Breibeck, Adam Serafin, Andreas Reichert, Stefan Maier, Bernhard Küster, Arne Skerra

**Affiliations:** 1 Munich Center for Integrated Protein Science, CIPS-M, and Lehrstuhl für Biologische Chemie, Technische Universität München, 85350 Freising-Weihenstephan, Germany; 2 Munich Center for Integrated Protein Science, CIPS-M, and Chair for Proteomics and Bioanalytics, Technische Universität München, 85354 Freising, Germany; 3 XL-protein GmbH, Lise-Meitner-Str. 30, 85354 Freising, Germany

**Keywords:** Electrospray ionization, PASylation, Protease cleavage, Recombinant polypeptide, Trypsin

## Abstract

We describe the design, preparation, and mass-spectrometric characterization of a new recombinant peptide calibration standard with uniform biophysical and ionization characteristics for mass spectrometry. “PAS-cal” is an artificial polypeptide concatamer of peptide cassettes with varying lengths, each composed of the three small, chemically stable amino acids Pro, Ala, and Ser, which are interspersed by Arg residues to allow site-specific cleavage with trypsin. PAS-cal is expressed at high yields in *Escherichia coli* as a Small Ubiquitin-like MOdifier (SUMO) fusion protein, which is easily purified and allows isolation of the PAS-cal moiety after SUMO protease cleavage. Upon subsequent in situ treatment with trypsin, the PAS-cal polypeptide yields a set of four defined homogeneous peptides in the range from 2 to 8 kDa with equal mass spacing. ESI-MS analysis revealed a conveniently interpretable raw spectrum, which after deconvolution resulted in a very simple pattern of four peaks with similar ionization signals. MALDI-MS analysis of a PAS-cal peptide mixture comprising both the intact polypeptide and its tryptic fragments revealed not only the four standard peptides but also the singly and doubly charged states of the intact concatamer as well as di- and trimeric adduct ion species between the peptides, thus augmenting the observable *m/z* range. The advantageous properties of PAS-cal are most likely a result of the strongly hydrophilic and conformationally disordered PEG-like properties of the PAS sequences. Therefore, PAS-cal offers an inexpensive and versatile recombinant peptide calibration standard for mass spectrometry in protein/peptide bioanalytics and proteomics research, the composition of which may be further adapted to fit individual needs.

**Figure:**
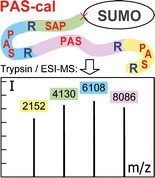
ᅟ

ᅟ

## Introduction

Modern mass spectrometers show ever improving performance and resolution. However, their use for increasingly demanding applications in proteomics research and bioanalytics necessitates reliable size standards for instrument calibration. Commonly employed standard reagents for ESI and MALDI mass spectrometry have to fulfil several requirements depending on the application, and they are expensive to produce.

Since in MALDI-MS singly charged analyte ions are predominantly formed and a large *m/z* range can be observed, it is reasonable to use large polymers for calibration, thus matching the expected size range. To this end, a large variety of chemical polymers [e.g., polyethylene glycol, poly(methylmethacrylate), polystyrene, polydimethylsiloxane, polystyrene sulfonate] [1–4], as well as peptides and proteins (e.g., bradykinin fragments, aldolase, insulin) [5] are commercially available. The chemical polymer calibration kits are optimized with regard to homogeneous ionization properties, but they comprise polydisperse substances with a poorly controlled distribution of molecular weights. In contrast, peptides and proteins constitute intrinsically monodisperse compounds, but it takes considerable effort to prepare different proteins in sufficient purity and to combine them at a suitable ratio and composition for MS applications. Furthermore, as the functionality of ionization sites in proteins is highly dependent on the chemical environment, which is defined by the amino acid sequence, use of conventional protein mixes as MS standards is hampered by uneven signal intensity distribution. Apart from this, synthetic peptides are limited in length by chemical solid phase procedures, and molecular weights above 3–4 kDa are hardly accessible at the required purity.

On the other hand, ESI-MS results in the formation of multiple-charged analyte ions with a characteristic *m/z* distribution in the raw spectrum. Usually, the molecular mass of an analyte becomes only apparent after mathematical deconvolution. The relatively narrow experimentally measured *m/z* range allows the use of rather small, chemically synthesized compounds for calibration, each of which appears as a singly charged molecule ion in the raw spectrum. In this regard, a collection of perfluorinated hydrocarbons (aliphatic and aromatic), trialkyl amines, as well as triazine [6] and phosphazene [7] derivatives, and also mixes thereof, are available, and even inorganic alkali salts may be used [8]. Amino compounds tend to carry desirable positive charges, whereas perfluorinated alkyl residues give rise to a mono-isotopic, simple mass increase of 50 Da per CF_2_ moiety. A substituted phosphazene compound with six fluoroalkyl side chains, for example, yields a signal distribution with a plain difference of 300 Da between the homologous compounds. Furthermore, perfluorinated alkyl chains are largely protected from secondary fragmentation reactions following electrospray ionization, which normally result in rapid degradation of nonfluorinated hydrocarbon chains of equivalent length. Nevertheless, the chemical synthesis of such compounds requires special effort and is costly [9].

A way to circumvent these problems would be a peptide standard with varying length but homogeneous composition, ideally prepared from a recombinant polypeptide comprising repetitive sequences of a small, defined set of amino acids. The PASylation technology, which was recently developed in our laboratory [10, 11], has inspired the design of an MS calibration standard that fulfils these requirements. PAS polypeptides are composed of long stretches of repetitive sequences of the three small, hydrophilic residues, proline, alanine and serine. These “PAS” sequences are natively disordered under physiological buffer conditions, and they adopt an expanded hydrodynamic volume, similar to the chemical polymer PEG. In contrast, they can be biosynthetically prepared using recombinant DNA technology. Initially, PAS sequences were developed for genetic fusion with therapeutic proteins, thus effecting retarded kidney filtration in vivo and resulting in a similar extending effect on the plasma half-life of biopharmaceuticals as chemical conjugation with PEG.

With regard to application as an MS standard, the lack of secondary structure of the PAS polypeptides and their exclusive composition of chemically stable amino acids without reactive side chains should result in a uniform ionization and protonation pattern for the peptide backbone, ensuring formation of multiple-charged ions especially suitable for ESI detection. Here, we report the design and synthesis of a PAS-based (poly)-peptide calibration standard which provides a simple *m/z* pattern, is easy to prepare, and should be useful for broad application in MS.

## Experimental

### PAS-cal Gene Construction

**Figure 1:**
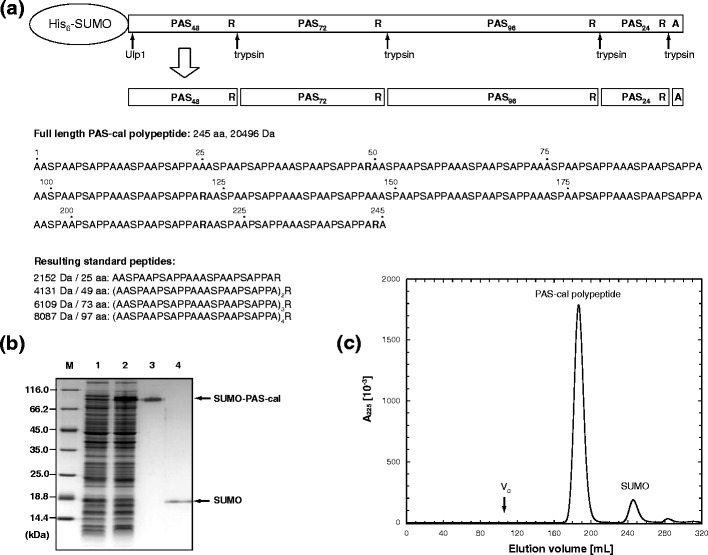
Construction, expression, and purification of SUMO/PAS-cal. (**a**) The PAS-cal polypeptide was expressed as a SUMO fusion protein. After protein purification and digestion with SUMO protease (Ulp1), the PAS-cal polypeptide concatamer yields a defined set of four standard peptides upon trypsin cleavage. (**b**) SDS-PAGE analysis of SUMO/PAS-cal purification. Lane M: low molecular weight marker; lane 1: uninduced *E. coli* cells; lane 2: induced cells after 3 h expression; lane 3: purified fusion protein; lane 4: SUMO protease digest of SUMO/PAS-cal. (**c**) Size exclusion chromatogram of the PAS-cal polypeptide after SUMO protease cleavage (applying the reaction mixture)

The assembly of a synthetic gene encoding the PAS-cal concatamer (Figure 1) was accomplished in several steps according to a previously developed strategy [12] involving incomplete ligation of synthetic DNA double strands that carry nonpalindromic three-nucleotide overhangs (corresponding to an Ala codon), followed by insertion into a plasmid that has a unique cleavage site for the type IIS restriction enzyme *Sap*I (5'-'NNN,NGAAGAGC). To this end, a pair of complementary oligodeoxynucleotides (Thermo Fisher Scientific, Ulm, Germany) encoding the 24-residue PAS#5 amino acid sequence [11]—with or without an additional Arg residue at the C-terminal end—was phosphorylated with polynucleotide kinase (PNK; Fermentas, St. Leon-Rot, Germany) and hybridized to obtain short gene cassettes, each with two 5'-overhangs of 3 nucleotides. These cassettes were ligated in the presence of a limiting amount of T4 DNA ligase (Fermentas). The resulting PAS-cal gene fragments, comprising two or three cassettes of either one or a mixture of both versions (i.e., with and without Arg), were extracted from a preparative agarose gel and subcloned on a derivative of pUC19 [13] that carries a double (inverse repeat) *Sap*I restriction site in its multiple cloning region, thus allowing both DNA sequence analysis and, subsequently, precise excision.

A derivative of the T7 expression vector pRSET5a [14, 15] with its multiple cloning site flanked by unique *Nde*I and *Hin*dIII restriction sites was used to insert the SUMO gene (*Smt3*). An additional *Sap*I recognition site within the pRSET5a backbone was removed via QuikChange site-directed mutagenesis (Stratagene, La Jolla, CA, USA) using primers 5'- GCGTATTGGGCGCTTCTCCGCTTCCTCGCTCAC-3' and 5'-GTGAGCGAGGAAGCGGAGAAGCGCCCAATACGC-3'. The structural gene for *Smt3*, which is devoid of introns, was amplified from chromosomal DNA of *S. cerevisiae* [16] using PCR primers 5'- ATCTAGCATATGAAACATCACCACCATCACCATTCGGACTCAGAAGTCAATCAAG-3' (forward) and 5'-CCTCATAAGCTTGCTCTTCAGGCGCCACCAATCTGTTC-3' (reverse), which also provided the sequences for an N-terminal His_6_-tag and a *Sap*I recognition site at the C-terminus. Suitable cloned PAS-cal gene fragments from above were then inserted into the single *Sap* I restriction site in a successive manner, finally resulting in the coding region for the fusion protein illustrated in Figure 1.

For expression of the recombinant truncated SUMO protease a gene fragment encoding the catalytic core of Ulp1 (aa 403–621) [17] was likewise amplified from *S. cerevisiae* chromosomal DNA by PCR using primers 5'-ATCTAGCATATGAAACATCACCACCATCACCATCTTGTTCCTGAATTAAATGAAAAAGACG-3' and 5'-CTTCATAAGCTTATTTTAAAGCGTCGGTTAAAATCAAATGGG-3', introducing an N-terminal His_6_-tag as above, followed by insertion into pRSET5a.

### PAS-cal Expression and Purification

The SUMO/PAS-cal fusion protein was expressed in the cytoplasm of *E. coli* BL21(DE3) [18] cotransformed with the expression vector and the plasmid pLysE to ensure efficient repression of basal promoter activity. Cells were grown at 37°C in a 2 L shake flask culture with Luria Bertani medium and induced with 0.5 mM isopropyl β-D-thiogalactopyranoside (IPTG) at OD_550_ ≈ 0.6 for 3 h. The cell pellet was homogenized in a French pressure cell (SLM Aminco, Urbana, IL, USA) and clarified by centrifugation and sterile filtration. The resulting protein solution was loaded onto a Ni/NTA resin (Ni Sepharose High Performance; GE Healthcare, Freiburg, Germany); then, an imidazole/HCl concentration gradient from 0 to 150 mM in 40 mM NaP_i_ pH 7.5, 500 mM NaCl was applied, and elution fractions were analyzed by SDS-PAGE. Selected fractions were combined and dialyzed against 20 mM Tris/HCl pH 9.5 and applied to a 1 mL Resource Q ion exchange column (GE Healthcare). Elution was performed with a NaCl concentration gradient from 0 to 200 mM in the same buffer, and selected fractions were combined and concentrated by ultrafiltration. Ten mg of SUMO/PAS-cal (final concentration: 1.7 mg/mL) was cleaved in the ion exchange running buffer with 10 μg of the recombinant truncated SUMO protease. This enzyme was produced in *E. coli* BL21(DE3) according to a published procedure [17], purified via IMAC and SEC, and stored in 1 mM β-mercaptoethanol, 50% (v/v) glycerol, 0.2% (v/v) Triton X–100 at –20°C prior to use.

After digest for 1 h at 37°C, the PAS-cal polypeptide was separated from the SUMO fragment and the protease by preparative SEC on a Superdex 200 26/60 column (GE Healthcare) in the presence of 50 mM NH_4_HCO_3_, monitoring UV absorption at 225 nm. Finally, for trypsin digestion, 30 μg of the PAS-cal polypeptide was first incubated with 0.5 μg trypsin (stock solution of 0.1 μg/mL sequencing grade modified trypsin in 50 mM acetic acid; Promega, Madison, WI, USA) at 37°C for 3 h, followed by addition of another 0.5 μg trypsin and continued incubation at 30°C overnight.

### ESI-MS

The trypsin-digested or intact PAS-cal polypeptide (0.28 mg/mL in 50 mM NH_4_HCO_3_) was supplemented with 20% (v/v) acetonitrile (LC-MS grade; Sigma-Aldrich, Steinheim, Germany) and 0.1% (v/v) formic acid (LC-MS grade; Sigma-Aldrich). Analysis was performed on an maXis Q-TOF mass spectrometer (Bruker Daltonics, Bremen, Germany) equipped with an ESI source (capillary voltage: 4.5 kV; end plate offset: –500 V; nebulizer pressure: 0.4 bar; dry gas flow: 4.0 L/min; dry temperature: 180°C). Raw data were analyzed and deconvoluted with Compass Data Analysis software (version 4.0). The observed *m/z* window was in the range of 500 to 3000 while best resolution was achieved between 500 and 1500. Electrospray Calibrant Solution (#63606-10ML; Fluka Analytical/Sigma Aldrich, Steinheim, Germany) was applied both for measuring the depicted ESI mass spectra and also in a side by side comparison with the PAS-cal calibration standard (see text). For the latter experiment, a sample of the recombinant antigen-binding fragment (Fab) of the antibody trastuzumab was prepared as previously described [19].

### MALDI-MS

Solutions of both the intact and trypsin-digested PAS-cal (0.28 mg/mL in 50 mM NH_4_HCO_3_) were mixed in a ratio 3:1 to trigger the formation of dimeric and trimeric peptide adduct ions in the MALDI spectrum. The resulting peptide mixture was supplemented with an equal volume of 2,5-dihydroxybenzoic acid (DHB, 10 μg/mL in 30% (v/v) acetonitrile in water, 0.2% (v/v) trifluoroacetic acid), and spotted onto a stainless steel target according to the dried droplet method. MALDI-MS analysis was performed on an UltrafleXtreme instrument (Bruker) in the linear positive mode (source voltage: 25 kV). Using 1000 shots per spectrum, an *m/z* range from 1000 to 23,000 was analyzed with ion suppression below *m/z* 1500; *m/z* values for the various expected peptide species were calculated based on the known amino acid sequences using Compass IsotopePattern software (version 1.3).

## Results and Discussion

To prepare a set of defined peptides for calibration purposes with sizes from 2 to 8 kDa—hence, extending the range of conventional chemical peptide synthesis—we have designed a recombinant PAS polypeptide concatamer that upon site-specific proteolysis yields four soluble ionizable peptides with a 2 kDa mass spacing, ideally suitable for MS analysis (Figure 1). After assembly from synthetic gene cassettes (see the Experimental section), this PAS-cal polypeptide was expressed in *E. coli* as an N-terminal SUMO-fusion protein [20], also equipped with a His_6_-tag, to ensure efficient translational initiation and accumulation in the bacterial cytoplasm [21].

After protein purification to homogeneity via immobilized metal affinity chromatography (IMAC) and ion exchange chromatography (IEC), the PAS-cal polypeptide was specifically cleaved from the fusion partner by means of SUMO protease Ulp1 [17, 22], and finally isolated via size exclusion chromatography (SEC). Each peptide cassette within the PAS-cal polymer is terminated by an Arg residue, thus allowing efficient trypsin cleavage and resulting in four peptide fragments in equimolar ratio. During the molecular design of PAS-cal, Arg was chosen instead of Lys since the more basic guanidinium group also provides one stable positive charge per peptide, thus ensuring high ion intensities in mass spectra obtained both by ESI and MALDI measurements [23].

The SUMO/PAS-cal fusion protein was produced in high yield of about 7 mg per L *E. coli* shake flask culture (Figure 1b), where it was found exclusively in the soluble cell extract, owing to the high inherent hydrophilicity of PAS polypeptides [10]. Due to the lack of hydrophobic side chains, the PAS concatamer was not stainable with Coomassie brilliant blue after cleavage from the fusion partner, but the progress of digest was visualized by the emerging low molecular weight protein band of the cleaved SUMO moiety (Figure 1b). The liberated PAS-cal polypeptide was detected during SEC as a peak at an absorption wavelength of 225 nm (i.e., by detecting the peptide backbone; see Figure 1c).

**Figure 2:**
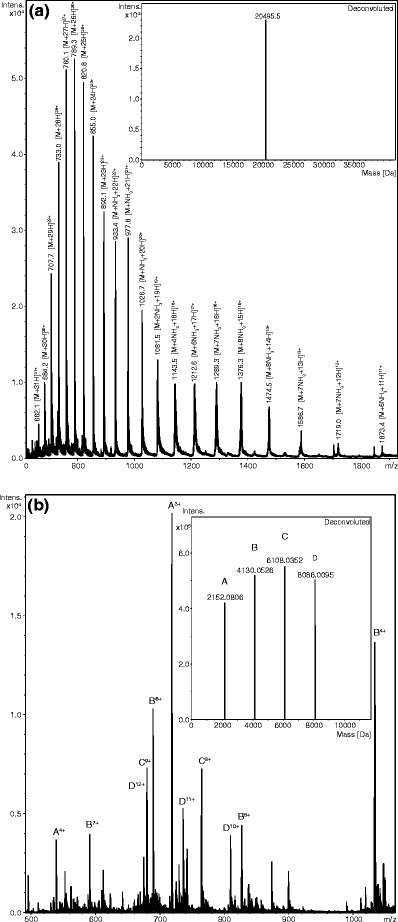
ESI-MS raw spectra of PAS-cal and its peptide fragments and deconvoluted spectra. (**a**) ESI-MS spectrum of the intact isolated PAS-cal polypeptide. The inset shows the deconvoluted spectrum. (**b**) ESI-MS spectrum of trypsin-digested PAS-cal with annotated *m/z* species. The inset shows the deconvoluted spectrum

The purified homogeneous PAS-cal polypeptide was subsequently digested with LC-MS grade trypsin in situ and directly applied to ESI-MS analysis on a Bruker maXis instrument (Figure 2). Indeed, a remarkably simple pattern of four peaks with masses of 2152.1, 4130.1, 6108.0, and 8086.0 Da was observed in the deconvoluted spectrum. The experimental data for both the intact concatamer (before tryptic digest) and the four resulting standard peptides perfectly matched the expected molecular masses (see Table 1 and Figure 2).

**Table 1:** ESI-MS Signals Measured for PAS-cal Peptides (Average Mass and *m/z* Numbers)

Species	Calculated mass [Da]	Deconvoluted mass [Da]	Observed*m/z* signals	
Peptide 1 (A)	2152.371	2152.0806	[M + 3H]^3+^	718.3800
			[M + 4H]^4+^	539.0368
Peptide 2 (B)	4130.541	4130.0526	[M + 4H]^4+^	1033.5390
			[M + 5H]^5+^	827.0327
			[M + 6H]^6+^	689.3618
			[M + 7H]^7+^	591.0254
Peptide 3 (C)	6108.711	6108.0352	[M + 8H]^8+^	764.5251
			[M + 9H]^9+^	679.6898
Peptide 4 (D)	8086.881	8086.0095	[M + 10H]^10+^	809.6224
			[M + 11H]^11+^	736.1119
			[M + 12H]^12+^	674.8532
PAS-cal (intact)	20495.536	20495.5	[M + 6NH_3_ + 11H]^11+^	1873.4
			[M + 7NH_3_ + 12H]^12+^	1719.0
			[M + 7NH_3_ + 13H]^13+^	1586.7
			[M + 8NH_3_ + 14H]^14+^	1474.5
			[M + 8NH_3_ + 15H]^15+^	1376.3
			[M + 7NH_3_ + 16H]^16+^	1289.3
			[M + 6NH_3_ + 17H]^17+^	1212.6
			[M + 4NH_3_ + 18H]^18+^	1143.5
			[M + 2NH_3_ + 19H]^19+^	1081.5
			[M + NH_3_ + 20H]^20+^	1026.7
			[M + NH_3_ + 21H]^21+^	977.8
			[M + NH_3_ + 22H]^22+^	933.4
			[M + 23H]^23+^	892.1
			[M + 24H]^24+^	855.0
			[M + 25H]^25+^	820.8
			[M + 26H]^26+^	789.3
			[M + 27H]^27+^	760.1
			[M + 28H]^28+^	733.0
			[M + 29H]^29+^	707.7
			[M + 30H]^30+^	684.2
			[M + 31H]^31+^	662.1

Interestingly, the longer the PAS-cal peptide fragment, the higher was the number of peaks in the raw spectra and also the intensity of the integrated MS signal, clearly indicating that not only the single Arg side chain and the N-terminal free amino group carry positive charges but that there are also contributions by “sliding protons” along the peptide backbone [24, 25]. This results in multiple-charged species—depending on the peptide length (see Table 1) —despite the lack of ionizable side chains within the PAS sequence itself. Nevertheless, the homogenous amino acid composition of the PAS (poly)peptides seems to favor a surprisingly even distribution and quality of ionization sites. This behavior is most likely a result of the disordered random coil structure of the PAS polypeptides [11]. This also explains the high accessibility for proteolytic cleavage during the preparation of the peptide mix. In fact, the deconvoluted ESI spectra of PAS-cal before and after tryptic digest demonstrate not only the high purity of the PAS-cal concatamer but also the absence of missed cleavage sites after proteolysis (Figure 2).

**Figure 3:**
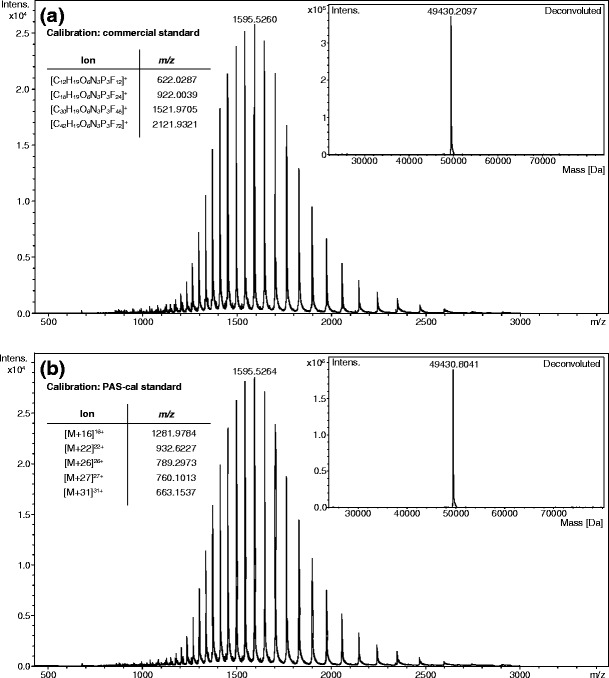
Comparison of ESI-MS raw and corresponding deconvoluted spectra of a purified recombinant protein sample measured after instrument calibration with (**a**) a commercial standard (Fluka electrospray calibrant solution) and (**b**) the PAS-cal polypeptide. The mono-isotopic *m/z* signals of each standard that were utilized for calibration are listed in the inset table. The most prominent *m/z* signals in the raw spectra of the sample protein are annotated whereas deconvoluted spectra are depicted in the insets. For comparison, the calculated average mass of the measured Fab fragment is 49429.6413 Da

To illustrate the use of PAS-cal as a calibrant for ESI-MS, we compared its applicability to a commercial calibration standard, followed by measuring the same protein sample, a 49 kDa recombinant Fab fragment, in two independent ESI experiments using a Bruker maXis Q-TOF instrument (Figure 3). In the first run, a commercially available fluoroalkyl phosphazene-based electrospray calibrant solution was used, whereas in the second measurement the mass spectrometer was calibrated with the intact PAS-cal, exploiting the resulting regular *m/z* pattern for stringent signal assignment. With both calibration methods, the deconvoluted average mass of the Fab fragment was very close (within ±1.2 Da) to the calculated mass of 49429.6 Da, which validates the use of PAS-cal for ESI calibration, demonstrating that large proteins can be measured accurately. The isolated PAS-cal polypeptide yielded stable *m/z* signals for several months when stored at –20°C and for at least a couple of weeks when kept at 4°C. Proteolytic degradation could not be observed as long as the biopolymer was maintained sterile.

**Figure 4:**
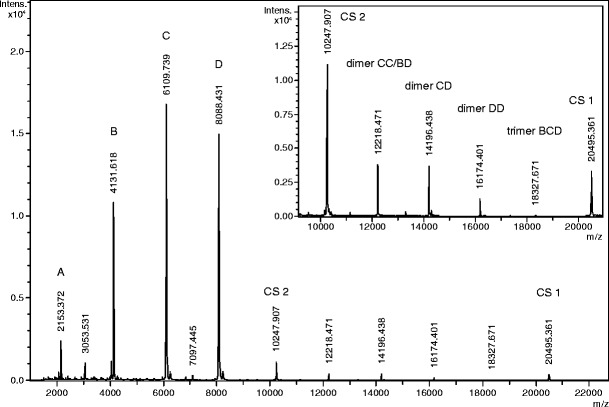
MALDI-MS spectrum of PAS-cal and its tryptic peptides acquired in linear mode. The inset shows an enlarged view of the charge states +1 and +2 of the intact polypeptide as well as the di- and trimeric species that are formed by the tryptic peptide fragments

In another application, MALDI-MS analysis of a PAS-cal peptide mixture comprising both the intact polypeptide and its tryptic peptide fragments revealed both the four standard peptides (A, B, C, D) and the singly (CS 1) as well as doubly (CS 2) charged states of the full-length concatamer at their expected *m/z* ratios (Figure 4 and Table 2). Notably, by adjusting the relative amount of the tryptic peptide fragments to a 3-fold excess over the intact PAS-cal, the formation of di- and trimeric adduct species among the peptides could be observed. It is well known from MALDI experiments that several peptides can “share” one charge by coordinating the same proton, leading to *m/z* ratios derived from the combined masses of the individual molecular species involved in the complex [26]. Most likely, the bridging protons are shared by the basic arginine side chains that are present in each PAS-cal peptide. Exploiting this phenomenon of adduct ion formation, it was possible to complement the *m/z* gap between the singly and doubly charged states of the intact polypeptide (see Table 2 and Figure 4). Note that due to the inherent sequence repetitivity of the concatamer, the *m/z* ratio 12218.5 can be assigned to two different dimeric peptide ion complexes. Apparently, the overlap of these two signals results in an enhanced intensity for this *m/z* ratio. These additional features of the PAS-cal standard in MALDI experiments make it even better suited for the calibration of a broad *m/z* range.

**Table 2:** MALDI-MS Signals Measured for PAS-cal Peptides (Average *m/z*)

Species		Calculated *m/z* signals	Observed *m/z* signals
Peptide 1 (A)	[M + H]^+^	2153.379	2153.372
Peptide 2 (B)	[M + H]^+^	4131.548	4131.618
Peptide 3 (C)	[M + H]^+^	6109.718	6109.739
Peptide 4 (D)	[M + H]^+^	8087.888	8088.431
PAS-cal (intact)	[M]^2+^ (CS 2)	10247.775	10247.907
	[M]^+^ (CS 1)	20495.536	20495.361
Dimer CC or BD	[M + M + H]^+^	12218.439	12218.471
Dimer CD	[M + M + H]^+^	14196.599	14196.438
Dimer DD	[M + M + H]^+^	16174.769	16174.401
Trimer BCD	[M + M + M + H]^+^	18327.147	18327.671

The use of an artificial polypeptide as MS calibration standard has been proposed before. The so-called QCAL [27], which is composed of 22 concatenated peptides that are liberated in uniform stoichiometry by tryptic digest, was designed to assess MALDI mass spectrometer performance for the separation and analysis of peptides. The cleaved peptides ought to yield a representative mixture of natural amino acids, including common sites for post-translational modifications, such as deamidation or methionine oxidation, to be optionally introduced by chemical treatment. QCAL was based on the QconCAT methodology previously developed in the same laboratory [28]. The concatameric QconCAT polypeptide contained a series of peptide fragments characteristic of proteins to be monitored in a proteomics study. If expressed under isotope-labeling conditions, the QconCAT peptide fragments were applicable as internal standard for quantification of the corresponding sample proteins by MS analysis. However, as the tryptic QCAL peptides fall into a rather low molecular weight range from 0.4 to 3.2 kDa, their suitability for MS calibration is generally more limited than with our PAS-cal standard.

Notably, both QconCAT and QCAL polypeptides were expressed in *E. coli* BL21(DE3) and occurred exclusively in the insoluble cell fraction [29]. This necessitated the use of 6 M guanidinium chloride to solubilize the inclusion bodies. The insolubility of the biosynthetic polypeptides also affected the efficiency of tryptic digest as not all predicted cleavage sites appeared to be equally accessible. In fact, only 10 of 20 predicted QconCAT peptide fragments could be assigned unambiguously in the mass spectra and, among those, the detectable analyte peptides were rather short (10 to 15 residues) [28]. In comparison, the 22 QCAL peptide fragments showed a broader range of lengths from 4 to 26 residues, but the closely similar molecular masses of some of the resulting peptides led to a narrow distribution of *m/z* ratios and complicated signal assignment. Some of the expected peptides could not be detected in ESI mass spectra or required chemical modification, like guanidination, to yield distinct signals [27]. These findings may be due to impaired ionization properties of some of the peptide fragments as the local protonation efficiency in the mass spectrometer strongly depends on the molecular structure.

In contrast, the PAS-cal peptides described here show uniform composition and are highly soluble, which does not only ensure strong ionization propensity but also allows efficient expression as a SUMO fusion protein as well as facile purification from the soluble cell extract, followed by quantitative protease cleavage. The ESI mass spectrum of the resulting peptides revealed a simple ensemble of signals, which all could be assigned unambiguously in the raw spectrum. By deconvolution the four expected molecular sizes for the set of PAS-cal peptides yield a broad mass range from 2 to 8 kDa with very similar intensities. This makes PAS-cal and its derived peptides a well-suited calibration standard, especially for the analysis of intact proteins. High mass accuracy is also an important goal in LC-MS experiments for proteomics studies, where peptide digest and extended measurements are usually involved. In this context, PAS-cal should provide a convenient “lock mass calibration” compound for spiking the actual sample, generating the calibration peptide mix with known and distinct *m/z* peaks at high signal intensities upon tryptic hydrolysis in situ. Thus, the variation between expected and measured *m/z* values can be used for calibration within the sample during prolonged measurement, while the PAS-cal peak pattern may serve at the same time to assess the completeness of peptide cleavage.

In general, the beneficial features of PAS-cal can be explained by the unique amino acid composition of PAS polypeptides. The designed mixture of the hydrophilic residues proline, alanine, and serine leads to a random coil conformation in aqueous solution that effects high solubility and excellent accessibility of trypsin cleavage sites, also providing uniform protonation sites at the amino and guanidine groups as well as along the peptide backbone. Apart from these useful properties for MS applications, long PAS polypeptides were successfully fused to biopharmaceutically active proteins without hampering their function in vitro or in vivo, which illustrates their biochemically inert behavior [10, 11].

## Conclusions

In this study, the biophysical advantages of the PASylation technology have been exploited to create a peptide calibration standard for mass spectrometry that shows (1) high expression yield in *E. coli* (even though just tiny amounts are needed for MS analysis), (2) high tryptic cleavage efficiency, and (3) well detectable signals with rather uniform intensities in ESI and MALDI mass spectra. Considering the flexible cloning strategy (see the Experimental section), the peptide standard range can be easily expanded or adjusted by inserting additional synthetic gene cassettes of desired lengths into the PAS-cal coding region. The broad mass range from 2 to 8 kDa covered by the present PAS-cal standard peptides makes them useful for calibration purposes in both ESI and MALDI applications. Preparation of the recombinant peptide standard may be further simplified by replacing the conformation-specific SUMO protease cleavage site with another Arg residue to allow liberation of all PAS peptides upon tryptic digest in situ. Apart from use as an isolated reagent, PAS-cal may also be fused to other proteins than SUMO, for example targets of biomedical interest. The resulting easily assignable MS signal signature should facilitate quantification in vivo, for example to measure intracellular expression levels in cell culture or plasma half-life in animal studies.
